# Comparison of the Japanese Orthopaedic Association (JOA) Score and Modified JOA (mJOA) Score for the Assessment of Cervical Myelopathy: A Multicenter Observational Study

**DOI:** 10.1371/journal.pone.0123022

**Published:** 2015-04-02

**Authors:** So Kato, Yasushi Oshima, Hiroyuki Oka, Hirotaka Chikuda, Yujiro Takeshita, Kota Miyoshi, Naohiro Kawamura, Kazuhiro Masuda, Junichi Kunogi, Rentaro Okazaki, Seiichi Azuma, Nobuhiro Hara, Sakae Tanaka, Katsushi Takeshita

**Affiliations:** 1 Department of Orthopaedic Surgery, the University of Tokyo, Tokyo, Japan; 2 Department of Orthopaedic Surgery, Yokohama Rosai Hospital, Yokohama, Japan; 3 Department of Spine and Orthopaedic Surgery, Japanese Red Cross Medical Center, Tokyo, Japan; 4 Department of Orthopaedic Surgery, Saitama Red Cross Hospital, Saitama, Japan; 5 Department of Orthopaedic Surgery, Musashino Red Cross Hospital, Musashino, Japan; University of Toronto, CANADA

## Abstract

**Objectives:**

The Japanese Orthopaedic Association (JOA) score is widely used to assess the severity of clinical symptoms in patients with cervical compressive myelopathy, particularly in East Asian countries. In contrast, modified versions of the JOA score are currently accepted as the standard tool for assessment in Western countries. The objective of the present study is to compare these scales and clarify their differences and interchangeability and verify their validity by comparing them to other outcome measures.

**Materials and Methods:**

Five institutions participated in this prospective multicenter observational study. The JOA and modified JOA (mJOA) proposed by Benzel were recorded preoperatively and at three months postoperatively in patients with cervical compressive myelopathy who underwent decompression surgery. Patient reported outcome (PRO) measures, including Japanese Orthopaedic Association Cervical Myelopathy Evaluation Questionnaire (JOACMEQ), the Short Form-12 (SF-12) and the Neck Disability Index (NDI), were also recorded. The preoperative JOA score and mJOA score were compared to each other and the PRO values. A Bland-Altman analysis was performed to investigate their limits of agreement.

**Results:**

A total of ninety-two patients were included. The correlation coefficient (Spearman’s rho) between the JOA and mJOA was 0.87. In contrast, the correlations between JOA/mJOA and the other PRO values were moderate (|rho| = 0.03 – 0.51). The correlation coefficient of the recovery rate between the JOA and mJOA was 0.75. The Bland-Altman analyses showed that limits of agreement were 3.6 to -1.2 for the total score, and 55.1% to -68.8% for the recovery rates.

**Conclusions:**

In the present study, the JOA score and the mJOA score showed good correlation with each other in terms of their total scores and recovery rates. Previous studies using the JOA can be interpreted based on the mJOA; however it is not ideal to use them interchangeably. The validity of both scores was demonstrated by comparing these values to the PRO values.

## Introduction

Cervical compressive myelopathy is a common disorder that frequently results in impairment of a patient’s motor, sensory and bladder function. Several scales that measure severity of physical disability have been developed to assess a patient’s pre- and post-treatment condition and the effectiveness of intervention. For example, the Japanese Orthopaedic Association (JOA) score was developed by the JOA in 1975. Since then, it has become one of the most frequently used outcome measures to evaluate functional status in patients with cervical myelopathy. Furthermore, and the concept of “recovery rate,” advocated by Hirabayashi et al., has been widely accepted as an outcome measure [1]. Currently, the revised version of the JOA score (1994), which includes an assessment of the shoulder and elbow function, is the most frequently used [2, 3].

One of the drawbacks of the JOA score is that it evaluates the degree of motor dysfunction by assessing a patient’s ability to use chopsticks. The use of chopsticks is limited to East Asian cultures including Japanese, Korean, Chinese and Vietnamese populations. The issues associated with using questionnaires related to cultural differences in eating methods have already been reported [4, 5]. Although chopsticks are now more widely used for eating, even in Western cultures, questionnaires using chopsticks cannot be readily applied to those who have not used them, or who do not use them regularly. Therefore, the adaptation of the JOA score to a Western population requires translation as well as modification [6]. Currently, there are three different kinds of so-called “modified JOA (mJOA) scores” [7–9]. However, the translation of these scores has not been validated and the scoring structure and content of evaluation items are substantially different. Despite their differences, the JOA score and the various modified scales are frequently confused with each other, and mistakenly discussed as being the same. Few comparisons of these scales have been made in the literature and few studies have assessed the validity of these scores. This causes confusion about which scale should be used in a certain population, and prevents us from comparing results of studies that used different modifications of the JOA score.

Therefore, it is very important to compare the properties of the JOA score and the mJOA score for the assessment of cervical myelopathy; the JOA score and the mJOA score. The objective of this study is to investigate the differences in and interchangeability of the JOA score and the mJOA score and to examine the validity of these scales by assessing correlations with other patient-reported outcome measures.

## Materials and Methods

The study protocol was approved by the institutional review board of the Clinical Research Support Center of the University of Tokyo Hospital. In order to secure a sufficient number of participants, we called for volunteers from our research group, “The University of Tokyo Spine Group,” and recruited five medical center institutions to participate in this prospective multicenter observational study. Ten surgeons in total were involved in this study. All eligible patients provided their written informed consent to participate in this study. All patients who underwent surgery for cervical compressive myelopathy between April 2013 and March 2014 were enrolled. Those with systemic diseases, including neurological disorders and rheumatoid arthritis, that could potentially affect motor function were excluded. Preoperatively, the surgeons recorded the following two scores.

### *JOA score* (Table A in S1 File) [2, 3]

We used the latest version of the JOA score in Japanese. This scale consists of six domain scores (motor dysfunction in the upper extremities, motor dysfunction in the lower extremities, sensory function in the upper extremities, sensory function in the trunk, sensory function in the lower extremities, and bladder function), scaled from 0 to 4, 4, 2, 2, 2, and 3, respectively, with the minimum total score being 0 and the maximum total score being 17. Yonenobu et al. defined the myelopathy severity as mild if the JOA score is larger than 13, as moderate if the JOA score ranges from 9 to 13 and as severe if the JOA score is less than 9 [3]. Motor function in the fingers was assessed based on the ability to use chopsticks and button clothing. Keller et al. published the modified version in German [9, 10]. The authors did not mention the use of cutlery, but rather simply used the term “fine motor function” for the assessment of motor function in the upper extremities. The score proposed by Chiles et al. is similar, although the authors mentioned the use of a knife and fork [8]. The recovery rate was calculated according to the following formula (Hirabayashi method): Recovery rate (%) = (postoperative JOA−preoperative JOA) / (17 [full score]—preoperative JOA) × 100 [1].

### *Modified JOA score (Benzel et al*.*)* (Table B in S1 File) [7]

This scale is the most commonly used among the so-called “mJOA” scores. Its scoring system differs from that of the original JOA in that it assesses only motor dysfunction in the upper and lower extremities, sensory function in the upper extremities, and bladder function, and does not include a scale for sensory function in the trunk and lower extremities. Each scale ranges from 0 to 7, 5, 3, and 3, respectively, with a total score of 0 to 18. Fehlings et al. defined the severity of myelopathy as mild if the mJOA score is 15 or larger, moderate if the mJOA score ranges from 12 to 14 or severe if the mJOA score is less than 12 [11]. This scale focused on the use of a spoon instead of chopsticks to evaluate motor function in the upper extremities. The recovery rate is calculated using the same formula as that applied for the original JOA, changing the full score from 17 to 18.

The differences between these scores are summarized in Table 1. The JOA score allocates 8 out of 17 (47%) points of the total score to motor function, while the mJOA score allocates 12 out of 18 (67%) points of the total score to motor function. These two scores were determined by the responsible surgeons at each institution. In addition to these scores, the Japanese Orthopaedic Association Cervical Myelopathy Evaluation Questionnaire (JOACMEQ) [12], Short Form-12 (SF-12) [13] and the Neck Disability Index (NDI) [14] were recorded as patient reported outcome (PRO) measurements. These scales were completed by the patients in the form of questionnaires. The postoperative scores were recorded whenever possible at follow-up visits performed three months after surgery.

**Table 1:** A summary of the differences between the JOA score and modified JOA score.

	Structure							Assessment of MU
	MU	ML	SU+	ST	SL	BL	Total	
JOA [3]	4	4	2	2	2	3	17	Chopsticks
Modified JOA [7]	5	7	3	N/A	N/A	3	18	Spoon

JOA: Japanese Orthopaedic Association score, MU: motor function in the upper extremities, ML: motor function in the lower extremities, SU: sensory function in the upper extremities, ST: sensory function in the trunk, SL: sensory function in the lower extremities, BL: bladder function, N/A: not applicable

The preoperative JOA and mJOA scores in each domain were compared with each other. The total scores were also compared with each other and to the PRO measurements. Furthermore, we compared the JOA and mJOA after dichotomizing the patients according to severity of motor function by the median of the JOA motor function scores. A prediction formula for the mJOA score was created using the JOA to enable direct comparisons between studies using these scores by converting the scores. We plotted the individual difference between the mJOA total score and the JOA total score (mJOA−JOA) against the average between the mJOA and JOA scores using a Bland–Altman plot. Bland-Altman analyses are now widely used for comparing two methods of measurement [15–19]. According to Bland and Altman, the limits of agreement can be estimated as the mean between duplicate measurements (the bias) ±1.96 SD, where the SD is the standard deviation of all of the paired differences [20]. This means that 95% of the differences will lie between these limits. Provided that differences within these lines are not clinically important, we could use the two measurement methods interchangeably. Although the minimally clinical important difference (MCID) of the JOA or mJOA has not been established, experts have argued that a difference of at least two points of mJOA is clinically important [21]. Therefore, the limits of agreement below 2 suggests the interchangeability of the two scores in the present study. Finally, among the patients whose postoperative scores at three months were available, the recovery rates for the JOA and mJOA scores were compared, and a Bland-Altman analysis was performed.

All analyses were carried out using the IBM SPSS Statistics Version 19 software package (SPSS, Inc., Somers, NY, USA). Correlations between the scores were analyzed by calculating the Spearman’s rank correlation coefficient rho. P-values less than 0.05 were considered to be significant in all statistical tests. We defined the strength of the correlation according to the general guideline (rho ≥ 0.70: very strong, ≥ 0.50: strong, ≥ 0.30: moderate, ≥ 0.10: weak) [22].

## Results

Ninety-two patients were included in the study. One patient whose bladder function could not be assessed due to anuria resulting from chronic renal failure was excluded. The mean age was 63.3 years (standard deviation: 12.9). The most common diagnosis indicated for surgery was cervical spondylotic myelopathy (58 patients), followed by ossification of the posterior longitudinal ligament (28 patients) and cervical disc herniation (six patients).

### Comparisons of the scores in each domain

The correlations between the JOA and mJOA scores in each domain were strong to very strong, with correlation coefficients of 0.84 for motor function in the upper extremities (p <0.001), 0.93 for motor function in the lower extremities (p <0.001), 0.67 for sensory function in the upper extremities (p <0.001) and 0.89 for bladder function (p <0.001). The correlation between the total scores for motor function (the sum of the scores for the upper and lower extremities) was also very strong (rho = 0.90, p <0.001).

### Total score

**Fig 1:**
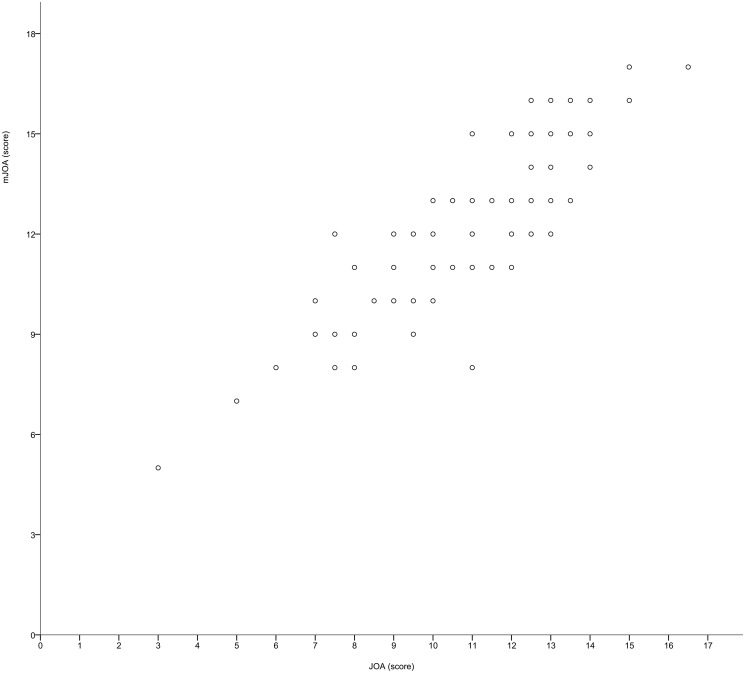
Scatterplot of the total scores for the JOA and mJOA scores (n = 92).

The mean preoperative JOA score was 11.2 (range: 3.0–16.5, standard deviation: 2.5), whereas the mean mJOA score was 12.4 (range: 5–17, standard deviation: 2.5). A scatterplot of the JOA and mJOA scores is shown in Fig 1, and the correlations between the preoperative scores are summarized in Table 2. The JOA and mJOA scores were very strongly correlated with each other (rho = 0.87, p <0.001). The median of the JOA motor function scores was 5. The correlation was found to be weaker in those with a motor function score less than 5 (n = 37, rho = 0.64) than in those with milder motor dysfunction (n = 55, rho = 0.77). On the other hand, the correlations between the JOA/mJOA scores and the other PRO values were not as strong. JOACMEQ QOL score, SF-12 PCS and NDI showed moderate correlations (|rho|: 0.41–0.51), whereas SF-12 MCS did not (|rho|: 0.03–0.05). While the very strong correlation between the JOA and mJOA scores demonstrates convergent validity, the moderate correlation with other PRO values suggests divergent validity. We created a prediction formula to calculate the total scores for the mJOA from the score of the JOA using linear regression analysis. The result is as follows:

mJOA total = 2.39+0.89×(JOA total)

The R^2^ of this equation was 0.78.

**Table 2:** Correlations between the preoperative total scores among the JOA, modified JOA, JOACMEQ QOL score, SF-12 PCS, MCS and NDI (n = 92).

	JOA	Modified JOA	JOACMEQ QOL	SF-12 PCS	SF-12 MCS	NDI
JOA	1	0.87*	0.41*	0.50*	-0.05	-0.50*
Modified JOA		1	0.41*	0.47*	0.03	-0.51*
JOACMEQ			1	0.29*	0.40*	-0.66*
SF-12 PCS				1	-0.29*	-0.47*
SF-12 MCS					1	-0.17
NDI						1

* Statistical significance

**Fig 2:**
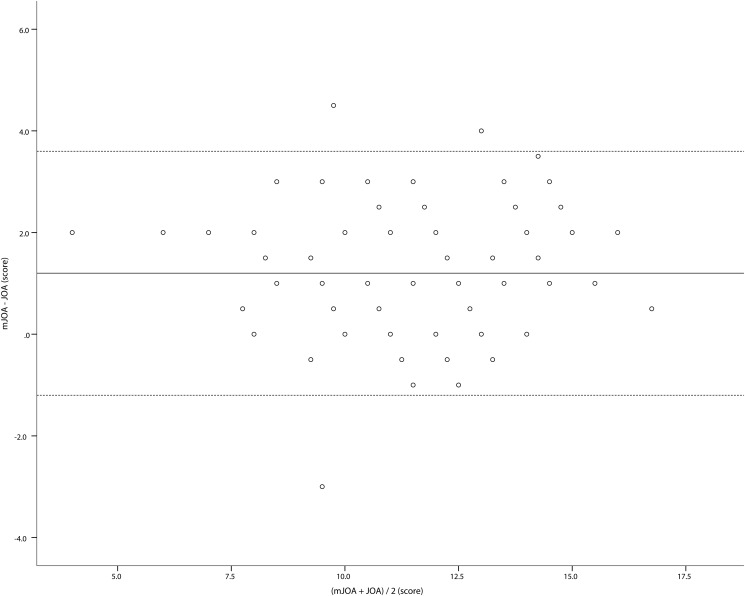
A Bland–Altman plot comparing the JOA and mJOA scores. The bias is shown as a solid line, and the upper and lower limits of agreement are shown as broken lines.

A Bland–Altman plot showing the differences between the two scores (mJOA−JOA) plotted against the mean of the two scores is shown in Fig 2. The mean difference between the two scores (the bias) was 1.2 (95% confidence interval: 0.9–1.5, standard deviation: 1.21). The upper and lower limits of agreement were 3.6 and -1.2, respectively. This range was well above the threshold we set based on an assumed MCID [21]; from this result, we were able to conclude that it is not ideal to interchange the JOA and mJOA.

### Recovery rate (RR)

**Fig 3:**
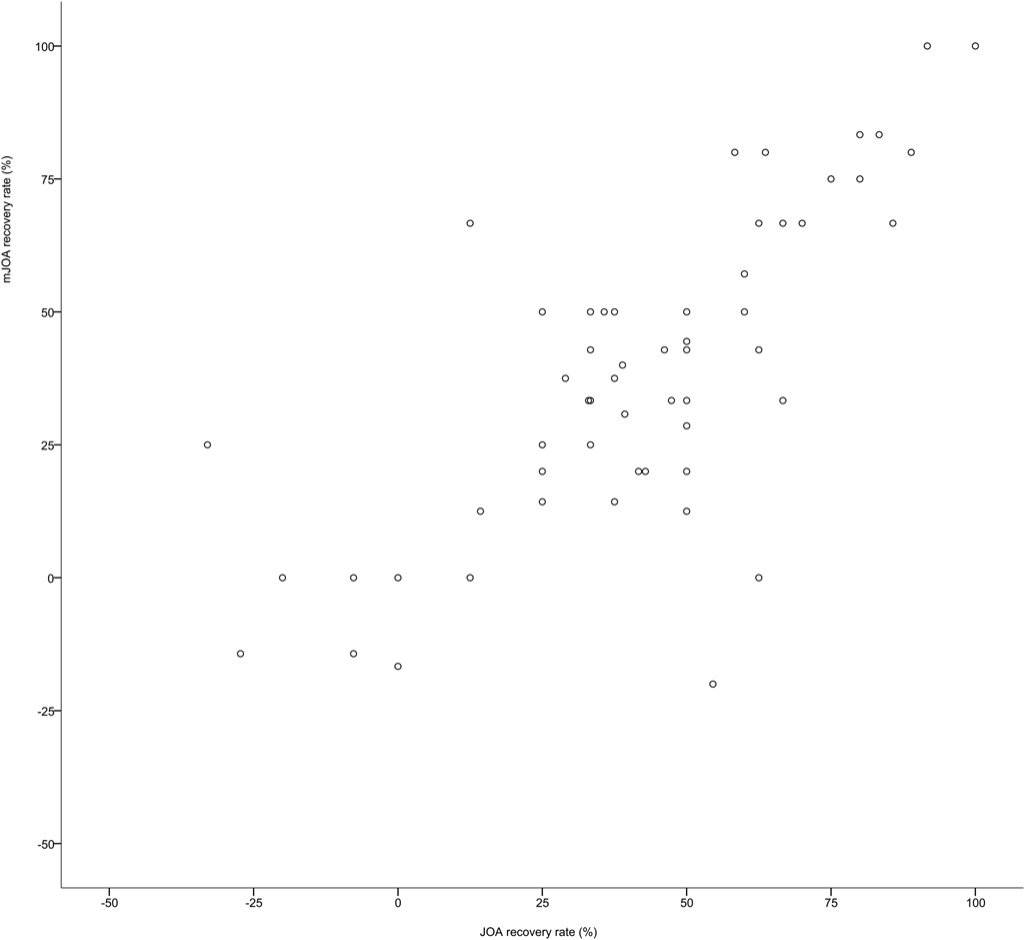
Scatterplot of the recovery rates for the JOA and mJOA scores. This figure includes only cases with a recovery rate from -1.0 to +1.0. Only two outliers were omitted (n = 63).

In 65 patients (71%) followed at three months postoperatively, the recovery rates were calculated using the Hirabayashi method and compared with each other. The mean JOA recovery rate was 45.1% (range: -33%– 100%, standard deviation: 30.8%), whereas the mean mJOA recovery rate was 38.2% (range: -200%– 100%, standard deviation: 43.0%). A scatterplot of the recovery rates for the JOA and mJOA is shown in Fig 3. In this figure, one outlier whose JOA RR was 0 and mJOA RR was -2.0 (deterioration), was omitted. Their correlations were very strong (rho: 0.75, p <0.001). In two cases, one scale showed recovery while the other showed deterioration. Both of these patients had urinary symptoms. We created a prediction formula to calculate the mJOA RR from the JOA RR using linear regression analysis. The result is as follows:

mJOA RR = -0.05+0.95×(JOA RR)

The R^2^ value of this equation was 0.46.

**Fig 4:**
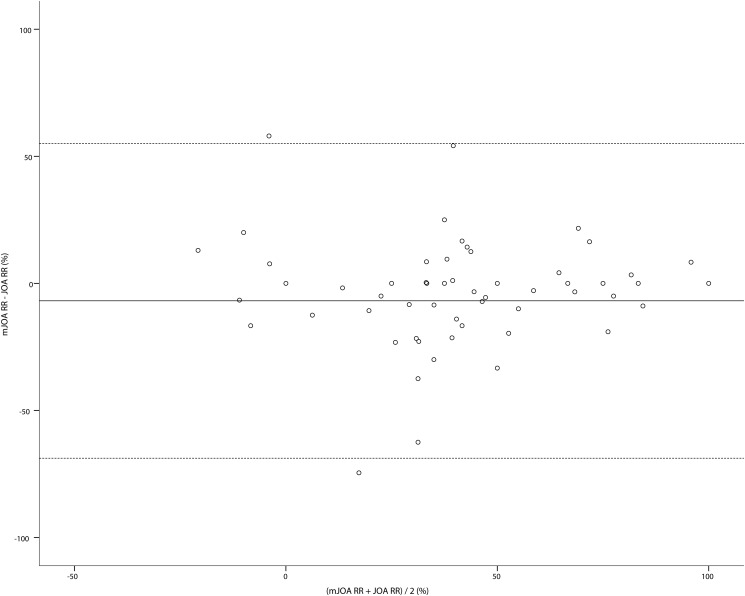
A Bland–Altman plot comparing the JOA and mJOA recovery rates. The bias is shown as a solid line, and the upper and lower limits of agreement are shown as broken lines.

A Bland–Altman plot showing the differences between the two recovery rates plotted against the mean of the two recovery rates is shown in Fig 4. The mean bias was -6.9% (95% confidence interval: -14.7%– 1.0%, standard deviation: 31.6%). The upper and lower limits of agreement were 55.1% and -68.8%, respectively. This range is also substantial enough to consider that it is not ideal to interchange the recovery rates of the JOA and mJOA.

## Discussion

There are two major findings in the present study. First, the domain and total scores of the JOA and mJOA were strongly correlated with each other. In addition, although the total scores and the recovery rates of the mJOA can be accurately predicted by the conversion formulas using the JOA score and its recovery rate, the Bland-Altman analyses showed they are not interchangeable. Second, the validity of the two types of JOA scores was demonstrated in comparisons with the PRO values.

No previous studies have directly compared the JOA and its modifications. The present study showed that the domain scores of the JOA and mJOA are strongly correlated, although the scoring structures of these scales differ in many domains, and the linearity of the scale is not guaranteed. It is of note that the mJOA score exhibited a very strong correlation with the JOA score, despite that the mJOA lacks scores for sensory function in the trunk and lower extremities. This finding may be due to the fact that severe sensory disturbances in the trunk or lower extremities are relatively rare in operative candidates for cervical myelopathy. The correlation between the scores for the sensory function in the upper extremities was lower than that for the other domains. This result may be explained by the exaggerated construct differences in which the JOA has two points and the mJOA has three points. The correlation in the subjects with severer motor dysfunction was weaker. This finding is also understandable given that the mJOA score gives a higher proportion to the motor function score. In the two patients with urinary symptoms, the recovery was not properly reflected in one scale. The bladder function score in the JOA tended to be exaggerated because the JOA criteria are more complicated than those of the mJOA. For example, the sense of urinary retention can lead to the patients receiving a score of 1, and this symptom is very common even in the elderly generation without myelopathy. These comparisons did not lead us to conclude that one scale had significant advantages over the other, and any of them can be used as desired based on the patients’ cultural background. The mJOA would be more easily accepted for Asian populations, since many of them now use a spoon, than would the JOA for Western populations, although no validated translations in Asian languages exist, and this would be an obstacle for raters who do not understand English. Using our conversion formulas, it is possible to interpret the results of previous studies that used the mJOA according to the original JOA score. For example, if a study set a certain cut-off point to evaluate the effectiveness of a treatment using the recovery rate, we speculate that the evaluation might be slightly stricter when using the mJOA instead of the JOA.

The present study showed that the scores for the mJOA and JOA are strongly correlated; however, Anscombe suggested that data with nearly identical simple statistical properties may appear very different when graphed [23]. A further understanding of the relationship between the mJOA and JOA scores can be achieved by looking at the differences between the two methods plotted against the mean score for each subject. We therefore examined the two seemingly compatible scores using Bland-Altman plots. Although Bland-Altman analyses were originally developed to make comparisons of two methods using the same scale, many authors have since applied this technique to the comparisons of two different scales [16, 19]. The range of the JOA score and mJOA score differ slightly (0–17 vs. 0–18), but very few patients in the present study achieved a nearly full score, which theoretically maximizes the difference between the two scales. Since a Bland-Altman analysis is the best method for visualizing errors and because there are no alternatives, we believe that the application of this method to the present dataset is acceptable. In Fig 2, the error appears unbiased, as differences are spread evenly and randomly above and below zero points. We examined the agreement between these two methods by looking at the spread of differences. The variability between the two methods is reflected by the limits of agreement, which were substantial in the present study. Based on this difference, a patient can easily be categorized into different groups of severity by both the JOA and mJOA.

While the criterion validity of the JOA score has been discussed by comparing it to the results of multiple other scales, including the Cooper myelopathy scale (CMS) [10, 24], European myelopathy scale (EMS) [10, 25] and Short Form-36 (SF-36) PCS [26, 27], few studies have discussed the validation of mJOA based on comparisons of these scores with the PRO values [28]. The mJOA score has been compared with the Nurick grade [29–31], NDI and SF-36 [31]. We measured the concurrent validity by performing comparisons to the JOACMEQ, SF-12 and NDI. In the present study, we used the SF-12 instead of the SF-36 because the summary scores for the SF-12 have been shown to mirror those of the SF-36 [13]. All of these results suggest divergent validity. The PRO forms are completed by the patients, as opposed to the JOA and mJOA, and these scales are substantially affected by the patients quality of life. Meanwhile, the JOA and mJOA are more disease-specific for cervical myelopathy and likely measure a different construct. These results are in accordance with the findings of the study by Kopjar et al. that validated the mJOA score [31].

There are some limitations associated with the present study. First, the rate of follow-up was not as high as expected. Many patients dropped out after the surgery as they were satisfied with their postoperative results. Therefore, the analysis of the recovery rate may have been biased. Second, because the assessment for the JOA score and mJOA were produced in different languages, the translational validity was not verified. Finally, the inter-observer reliability and test-retest reliability were not investigated in the present study. However, the inter- and intra- observer reliability of the JOA is reported to be high [3]. The inter-observer reliability of the mJOA has also been reported to be high [32], although this finding should be interpreted with caution since a translated version of the scale was used in this study. Unfortunately, the test-retest reliability of the mJOA has not yet been established. Further studies may make it possible to compare the properties of these scores.

## Conclusion

In conclusion, the mJOA score is very strongly correlated with the JOA, and previous studies using the JOA can be interpreted based on the mJOA based on this speculation, especially by using the conversion formulas advocated in this report. However, the Bland-Altman analysis revealed that it is not ideal to use these scoring systems interchangeably.

## Supporting Information

S1 Dataset Dataset of the outcomes in the participants. (XLSX)

S1 File Table A, Japanese Orthopaedic Association Score (English translation) [3]. **Table B,** Modified Japanese Orthopaedic Association Score [7].
(DOCX)
