# Correction: Comparison of the Japanese Orthopaedic Association (JOA) Score and Modified JOA (mJOA) Score for the Assessment of Cervical Myelopathy: A Multicenter Observational Study

**DOI:** 10.1371/journal.pone.0128392

**Published:** 2015-05-11

**Authors:** The PLOS ONE Staff

There is an error in Table 1. The heading in column 3 should be “SU.” Please see the corrected Table 1 here. The publisher apologizes for the error.

**Table 1:** A summary of the differences between the JOA score and modified JOA score.

	Structure							Assessment of MU
	MU	ML	SU	ST	SL	BL	Total	
JOA [3]	4	4	2	2	2	3	17	Chopsticks
Modified JOA [7]	5	7	3	N/A	N/A	3	18	Spoon

JOA: Japanese Orthopaedic Association score, MU: motor function in the upper extremities, ML: motor function in the lower extremities, SU: sensory function in the upper extremities, ST: sensory function in the trunk, SL: sensory function in the lower extremities, BL: bladder function, N/A: not applicable
