# Supplementary material for: Comparison of the Japanese Orthopaedic Association (JOA) Score and Modified JOA (mJOA) Score for the Assessment of Cervical Myelopathy: A Multicenter Observational Study
Source: PLoS One. 2015 Apr 2;10(4):e0123022. doi: 10.1371/journal.pone.0123022 (PMC4383381; doi:10.1371/journal.pone.0123022)
Supplement: S1 File — Table B, Modified Japanese Orthopaedic Association Score [7]. (DOCX) [file pone.0123022.s002.docx]

**Table A.** Japanese Orthopaedic Association Score (English translation) [3].

| Motor function | | | |  |  |  |  |  |
| --- | --- | --- | --- | --- | --- | --- | --- | --- |
|  | Fingers | |  |  |  |  |  |  |
|  |  | 0 | Unable to feed oneself with any tableware including chopsticks, spoon, or fork, and/or unable to fasten buttons of any size | | | | | |
|  |  | 1 | Can manage to feed oneself with a spoon and/or fork but not with chopsticks | | | | | |
|  |  | 2 | Either chopstick-feeding or writing is possible but not practical, and/or large buttons can be fastened | | | | | |
|  |  | 3 | Either chopstick-feeding or writing is clumsy but practical, and/or cuff buttons can be fastened | | | | | |
|  |  | 4 | Normal |  |  |  |  |  |
|  | Shoulder and elbow (evaluated by MMT score of the deltoid or biceps muscles, whichever is weaker) | | | | | | | |
|  |  | -2 | MMT 2 or less | |  |  |  |  |
|  |  | -1 | MMT 3 |  |  |  |  |  |
|  |  | -0.5 | MMT 4 |  |  |  |  |  |
|  |  | 0 | MMT 5 |  |  |  |  |  |
|  | Lower extremity | | |  |  |  |  |  |
|  |  | 0 | Unable to stand up and walk by any means | | | |  |  |
|  |  | 0.5 | Able to stand up but unable to walk | | | |  |  |
|  |  | 1 | Unable to walk without a cane or other support on a level | | | | | |
|  |  | 1.5 | Able to walk without support but with a clumsy gait | | | | |  |
|  |  | 2 | Walks independently on a level but needs support on stairs | | | | | |
|  |  | 2.5 | Able to walk independently when going upstairs, but needs support when going downstairs | | | | | |
|  |  | 3 | Capable of fast but clumsy walking | | | |  |  |
|  |  | 4 | Normal |  |  |  |  |  |
| Sensory function | | | |  |  |  |  |  |
|  | Upper extremity | | |  |  |  |  |  |
|  |  | 0 | Complete loss of touch and pain sensation | | | |  |  |
|  |  | 0.5 | 50% or less normal sensation and/or severe pain or numbness | | | | | |
|  |  | 1 | More than 60% normal sensation and/or moderate pain or numbness | | | | | |
|  |  | 1.5 | Subjective numbness of slight degree without any objective sensory deficit | | | | | |
|  |  | 2 | Normal |  |  |  |  |  |
|  | Trunk | |  |  |  |  |  |  |
|  |  | 0 | Complete loss of touch and pain sensation | | | |  |  |
|  |  | 0.5 | 50% or less normal sensation and/or severe pain or numbness | | | | | |
|  |  | 1 | More than 60% normal sensation and/or moderate pain or numbness | | | | | |
|  |  | 1.5 | Subjective numbness of slight degree without any objective sensory deficit | | | | | |
|  |  | 2 | Normal |  |  |  |  |  |
|  | Lower extremity | | |  |  |  |  |  |
|  |  | 0 | Complete loss of touch and pain sensation | | | |  |  |
|  |  | 0.5 | 50% or less normal sensation and/or severe pain or numbness | | | | | |
|  |  | 1 | More than 60% normal sensation and/or moderate pain or numbness | | | | | |
|  |  | 1.5 | Subjective numbness of slight degree without any objective sensory deficit | | | | | |
|  |  | 2 | Normal |  |  |  |  |  |
| Bladder function | | | |  |  |  |  |  |
|  |  | 0 | Urinary retention and/or incontinence | | | |  |  |
|  |  | 1 | Sense of retention and/or dribbling and/or thin stream and/or incomplete continence | | | | | |
|  |  | 2 | Urinary retardation and/or pollakiuria | | | |  |  |
|  |  | 3 | Normal |  |  |  |  |  |

**Table B.** Modified Japanese Orthopaedic Association Score [7].

| Motor dysfunction score of the upper extremities | | |
| --- | --- | --- |
|  | 0 | Inability to move hands |
|  | 1 | Inability to eat with a spoon, but able to move hands |
|  | 2 | Inability to button shirt, but able to eat with a spoon |
|  | 3 | Able to button shirt with great difficulty |
|  | 4 | Able to button shirt with slight difficulty |
|  | 5 | No dysfunction |
|  |  |  |
| Motor dysfunction score of the lower extremities | | |
|  | 0 | Complete loss of motor and sensory function |
|  | 1 | Sensory preservation without ability to move legs |
|  | 2 | Able to move legs, but unable to walk |
|  | 3 | Able to walk on flat floor with a walking aid (i.e., cane or crutch) |
|  | 4 | Able to walk up and/or down stairs with hand rail |
|  | 5 | Moderate to significant lack of stability, but able to walk up and/or down stairs without hand rail |
|  | 6 | Mild lack of stability but walks with smooth reciprocation unaided |
|  | 7 | No dysfunction |
|  |  |  |
| Sensory dysfunction score of the upper extremities | | |
|  | 0 | Complete loss of hand sensation |
|  | 1 | Severe sensory loss or pain |
|  | 2 | Mild sensory loss |
|  | 3 | No sensory loss |
|  |  |  |
| Sphincter dysfunction score | | |
|  | 0 | Inability to micturate voluntarily |
|  | 1 | Marked difficulty with micturition |
|  | 2 | Mild to moderate difficulty with micturition |
|  | 3 | Normal micturition |
